# Differentiated effects of medical educational collaboration on primary care employment intention: a multi-group analysis based on competence types

**DOI:** 10.3389/fmed.2026.1789864

**Published:** 2026-05-14

**Authors:** Yang Rong

**Affiliations:** 1 Yunnan Normal University, Kunming, Yunnan, China; 2 Kunming University, Kunming, Yunnan, China

**Keywords:** competence profiles, medical educational collaboration, multi-group analysis, primary care employment intention, social cognitive career theory

## Abstract

This study aimed to investigate the differential associations between medical educational collaboration and primary care employment intention among medical students with distinct competence profiles, drawing upon Social Cognitive Career Theory (SCCT) as the theoretical framework. A cross-sectional survey was conducted among 398 clinical medicine students from three medical schools in Yunnan Province, China. Latent profile analysis was employed to classify participants into competence profiles based on self-assessed clinical reasoning, communication skills, and procedural skills. Multi-group structural equation modeling was performed to examine whether the SCCT-based pathways from medical educational collaboration to primary care employment intention varied across profiles. Three competence profiles were identified: Practice-Oriented (35.7%), Balanced (33.7%), and Reasoning-Oriented (30.6%). The overall structural model demonstrated acceptable fit, with career self-efficacy and outcome expectations serving as parallel and sequential mediators, with approximately 66% of the total association transmitted through indirect pathways. The multi-group analysis revealed significant pathway heterogeneity across profiles. For Practice-Oriented students, outcome expectations served as the dominant mediating pathway, while the association between career self-efficacy and primary care employment intention was non-significant. For Reasoning-Oriented students, career self-efficacy played a more critical mediating role, with the strongest association with primary care employment intention among the three profiles. The Balanced profile exhibited relatively equal contributions from both pathways. These findings provide empirical evidence suggesting that SCCT-based pathways may vary as a function of individual competence characteristics, contributing to the person-centered perspective in career development research. The results suggest that medical education institutions should implement differentiated career guidance interventions tailored to students’ competence profiles to strengthen the primary care workforce.

## 1 Introduction

The global healthcare system faces an unprecedented crisis in primary care workforce distribution, with an estimated shortage of 10 million healthcare workers projected by 2030, predominantly affecting low and middle income countries (1). In the developed countries, there is burnout among the family physicians, which is linked to the intention to leave practice and the quality of patient care provided (2). Apart from burnout, this problem of healthcare maldistribution also affects rural areas, as the rural population is served by only 10% of the total number of physicians, despite the fact that 20% of the American population lives in rural areas (3). This problem of healthcare maldistribution is a concern for the healthcare systems around the world.

China confronts similar challenges in strengthening its primary healthcare workforce, particularly in western and rural regions (4). The central government has formulated many policies to address the issue of human resource shortage in primary healthcare, realizing that the shortage of qualified general practitioners is the biggest challenge to the implementation of the tiered diagnosis and treatment system (5). The “5 + 3” system for training medical professionals was put forward in 2017 by the State Council’s plan for the reform of medical education and the integration of medical education and clinical practice. This plan laid the groundwork for the Healthy China 2030 strategy (6). The Healthy China Action Plan (2019–2030) also underlines disease prevention, health promotion, and the construction of a universal basic healthcare system to provide safe, effective, and affordable primary care services (7). In spite of these initiatives, it is difficult to attract medical graduates to work in grassroots institutions in terms of monetary gain, career growth opportunities, and work environment (8).

Identifying the determinants of medical students’ career plans with respect to the practice of primary health care has emerged as a prominent area of research interest. Several factors have been established to influence rural and primary healthcare career plans, including rural origin, experiences with clinical rotations, perceptions of compensation, and opportunities for professional development (9). Studies have found that factors like exposure to education, personal interest, lifestyle factors, and awareness of the job requirements play a significant role in influencing the preferences for specialties and location of practice (10). The choice of general practice has been found to relate to the training experience and previous positive exposure to the specialty (11).

Social Cognitive Career Theory (SCCT) developed by Lent, Brown, and Hackett is a strong theoretical model for explaining processes involved in career development (12). The theory posits that three cognitive-person variables—self-efficacy beliefs, outcome expectations, and personal goals—interact dynamically with contextual and experiential factors to shape career interests, choices, and performance (13). Self-efficacy, which refers to individuals’ belief in the ability to perform career-related activities, is found to be a strong predictor for medical students’ career choices in different specialties (14). Outcome expectations refer to individuals’ anticipated beliefs about the consequences of engaging in particular career-related behaviors—that is, the perceived likelihood that pursuing a given career path will yield valued outcomes such as material rewards, social recognition, or personal fulfillment (12). Personal goals, the third core construct, represent individuals’ intentions to engage in a specific activity or to attain a particular career outcome (12); in the present study, this construct is operationalized as primary care employment intention, defined as the intention to seek employment in primary care settings upon graduation. Under the SCCT framework, learning experiences, which include training, guidance, and academic environments, are crucial sources that influence the development of self-efficacy and career intentions (15). The application of SCCT to medical education contexts has gained increasing scholarly attention, with researchers developing domain specific scales to assess career intentions toward particular specialties (16).

Medical educational collaboration, as a comprehensive reform initiative integrating medical education with clinical practice, involves a broad spectrum of aspects, such as the quality of clinical placements, the nature of the teaching hospital environment, and the role of mentor support (17). Within the SCCT framework, these aspects constitute proximal learning experiences that shape students’ self-efficacy beliefs and outcome expectations, and thereby their primary care employment intention (12). However, it should be noted that previous studies have mainly explored the macro-level implications of such educational experiences, and insufficient attention has been paid to individual differences. Little has been explored about whether the SCCT-based pathways from medical educational collaboration to primary care employment intention vary for students with different competence levels in such areas as clinical practice, research, and communication.

This study attempts to explore how medical education collaboration may be differentially associated with primary care employment intention among medical students in Yunnan Province, China. By means of a multi-group analysis grounded in competence types, it is attempted to clarify how medical students with different competence profiles respond to medical education collaboration experience and how such experiences are associated with the development of primary care employment intention. It is expected that the findings will contribute to the refinement of career development theories and provide practical implications for the development of interventions that would improve the primary health care workforce in western China.

## 2 Materials and methods

### 2.1 Research design

The study used a cross-sectional survey design to investigate the differential associations between collaboration in medical education and medical students’ primary care employment intention. Based on the theory of Social Cognitive Career Theory (SCCT), which posits that learning outcomes shape career intentions by influencing self-efficacy and outcome expectations, the study used a conceptual framework to investigate the associations between collaboration in medical education and primary care employment intention with self-efficacy and outcome expectations as mediators. A multi-group structural equation model analysis was used to determine if the pathways differed significantly among students with different levels of competence. The survey was conducted in Yunnan Province, China between March and October 2024. This study received ethical approval from the Ethics Committee of Tourism School of Kunming University (Approval Number: YNNU-2024-IRB-045). All participants provided informed consent prior to data collection, and all responses were anonymized to ensure confidentiality.

### 2.2 Participants and sampling

The target population was undergraduate clinical medicine students enrolled in 5-year programs at medical schools in Yunnan Province. Fourth-year and fifth-year students were chosen because they have completed a significant number of clinical rotations that would allow them to make valid assessments of cooperation in medical education. A stratified cluster sampling method was used. The three medical schools selected for the study represented different types of institutions: a provincial key medical university, a regional comprehensive university that houses a medical school, and a science and engineering university that provides a medical education. This ensured diversity in terms of institutional history, allocation of resources, and orientation of education. Within each institution, classes were randomly chosen as sampling units, and all eligible students in these classes were approached to participate.

The inclusion criteria were: (1) full-time undergraduate students majoring in clinical medicine; (2) enrolled in the fourth or fifth year of study; (3) having completed at least one semester of clinical rotation; and (4) willing to participate voluntarily. Students were ineligible for inclusion in the study if they were enrolled in non-clinical programs in medicine, had not yet begun their clinical education, or were on leave of absence during the survey period.

**FIGURE 1:**
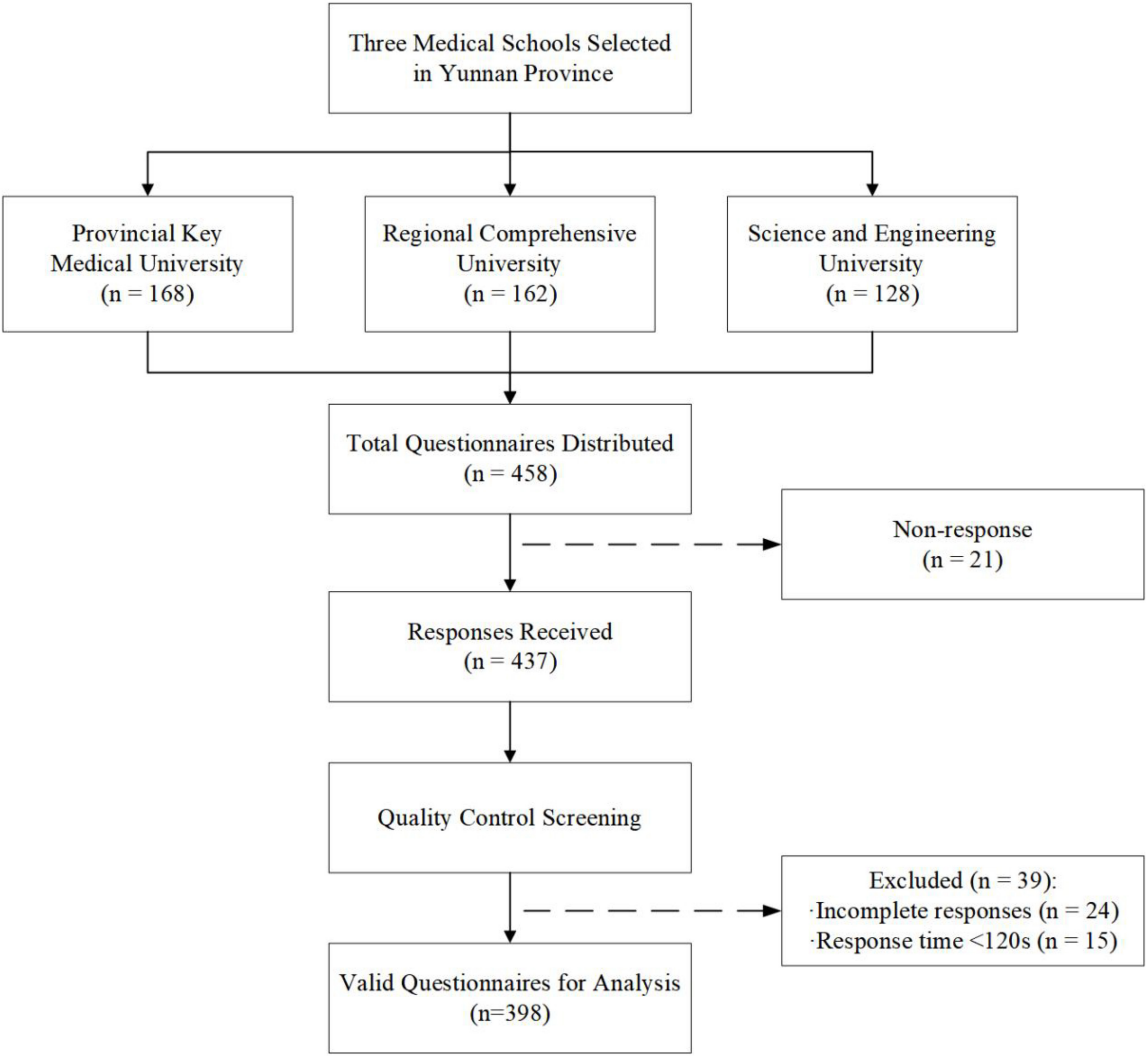
Flowchart of participant recruitment and data screening process.

A structured self-reporting online questionnaire was designed and administered through the Wenjuanxing platform. Prior to conducting the formal study, a pilot test was carried out among 30 students to examine the clarity of items and overall length of the research tool. Some modifications were made based on their response during the data collection procedure. Each device was restricted to one submission through IP address verification to prevent duplicate responses. Attention check items were embedded within the questionnaire to identify careless responding. The platform automatically recorded response timestamps, allowing identification of responses completed too quickly to reflect genuine consideration. As shown in Figure 1, a total of 458 questionnaires were dispatched to the three universities. These included 168 from the provincial key medical university, 162 from the regional comprehensive university, and 128 from the science and engineering university. A total of 437 were received. After conducting the quality control procedures, 24 were considered to be invalid because they were not completed correctly. Additionally, 15 were considered invalid because they took less than 120 s to complete. The final analytic sample consisted of 398 valid questionnaires, yielding an effective response rate of 86.9%.

### 2.3 Instruments

In order to examine medical educational collaboration, a scale was designed based on the conceptual framework of medical educational collaboration in China. This scale included 15 items, which covered three dimensions: clinical teaching quality (5 items), institutional support (5 items), and career guidance (5 items). Sample items included “My clinical teachers effectively integrate theoretical knowledge with practical skills” and “The hospital provides adequate opportunities for hands-on clinical practice.” The responses were measured on a five-point Likert scale ranging from 1 (strongly disagree) and 5 (strongly agree). The Cronbach’s alpha coefficient for the total scale was 0.91, with subscale alphas ranging from 0.84 to 0.88.

Career self-efficacy was measured using the Career Decision Self-Efficacy Scale-Short Form (18). This scale contains 25 items that relate to five factors: self-appraisal, occupational information, goal selection, planning, and problem solving. The scale uses a five-point Likert scale that ranges from 1 (no confidence at all) to 5 (complete confidence). The Chinese version of the scale was found to have high reliability and validity when used in previous research with medical students (19). In this study, the Cronbach’s alpha was 0.93.

Outcome expectations about career outcomes in primary care were measured using a 12-item scale developed based on the Social Cognitive Career Theory outcome expectations framework (20). This scale measured the expected outcomes of having a career in primary care in the areas of material returns (4 items), social recognition (4 items), and personal fulfillment (4 items). Sample items included “Working in primary care would provide me with a stable income” and “Working in primary care would allow me to make meaningful contributions to community health.” Responses ranged from 1 (strongly disagree) to 5 (strongly agree) with the Cronbach’s alpha being 0.89.

Primary care employment intention was measured with a 4-item scale designed for this study. These items included the participants’ intention to work in community health centers, township health centers, and village clinics after graduation. A sample item was “I intend to seek employment in a primary care setting upon graduation.” Responses were recorded on a five-point Likert scale from 1 (strongly disagree) to 5 (strongly agree) with the Cronbach’s alpha being 0.86.

To classify the participants into different competency profiles, a 10-item self-report assessment of clinical competency was administered. This included competency related to clinical reasoning (4 items), communication skills (3 items), and procedural skills (3 items). The Cronbach’s alpha for the total scale was 0.85. Latent profile analysis was then performed on the competency groups to generate a grouping variable that was used for multi-group structural equation modeling.

### 2.4 Statistical analysis

Descriptive statistics were calculated for all variables, including means, standard deviations, frequencies, and percentages. Independent samples *t*-tests and one-way analysis of variance (ANOVA) were carried out to examine differences in primary care employment intention. Pearson correlation coefficients were computed among the main study variables, and variance inflation factors (VIF) were examined to assess potential multicollinearity. To assess the potential for common method bias arising from the single-source self-report design, Harman’s single-factor test was conducted by entering all study items into an exploratory factor analysis and examining the variance explained by the first unrotated factor. The first factor accounted for 32.4% of the total variance, falling well below the recommended threshold of 50%, suggesting that common method bias was unlikely to pose a serious threat to the validity of the findings (21). Confirmatory factor analysis (CFA) was carried out for examining measurement models for medical educational collaboration, career self-efficacy, outcome expectations, and primary care employment intention. Model fit was assessed using multiple indices, including chi-square to degrees of freedom ratio (χ^2^/df < 3), comparative fit index (CFI > 0.90), Tucker-Lewis index (TLI > 0.90), root mean square error of approximation (RMSEA < 0.08), and standardized root mean square residual (SRMR < 0.08) (22). Convergent validity was evaluated through factor loadings (> 0.50) and average variance extracted (AVE > 0.50), while discriminant validity was assessed by comparing the square root of AVE with inter-construct correlations (23). Latent profile analysis (LPA) was employed to identify distinct subgroups of students based on their clinical competence self-assessment scores. Models with two to five latent profiles were estimated and compared using the Akaike Information Criterion (AIC), Bayesian Information Criterion (BIC), sample-size adjusted BIC (aBIC), entropy, and the Lo-Mendell-Rubin likelihood ratio test (LMR-LRT) (24). Multi-group structural equation modeling was used to test the proposed associations between medical educational collaboration, career self-efficacy, outcome expectations, and primary care employment intention for the specified competence profiles. Measurement invariance was examined through a sequence of configural, metric, and scalar invariance models (25). Path coefficients were compared across groups to investigate whether the associations between medical educational collaboration and primary care employment intention differed depending on the competence profile. The mediation effect was assessed using bias-corrected bootstrap confidence intervals with 5,000 resamples, and the mediation effect was considered significant if the 95% confidence interval did not include zero (26). All analyses were performed using SPSS 26.0 for descriptive statistics and the Harman’s single-factor test, and Mplus 8.3 for CFA, LPA, and multi-group SEM. All Mplus models were estimated using maximum likelihood estimation with robust standard errors (MLR) to account for potential non-normality in the Likert-scale response data.

## 3 Results

### 3.1 Descriptive statistics and competence profile classification

The demographic characteristics of the 398 participants are presented in Table 1. The sample consisted of 156 males (39.2%) and 242 females (60.8%), with 189 fourth-year students (47.5%) and 209 fifth-year students (52.5%). More than half of the participants (57.0%) reported rural household registration, and the majority (71.9%) had parents with high school education or below. The mean scores for the main study variables were as follows: medical educational collaboration (*M* = 3.56, SD = 0.72), career self-efficacy (*M* = 3.41, SD = 0.68), outcome expectations (*M* = 3.28, SD = 0.81), and primary care employment intention (*M* = 2.87, SD = 0.94). Independent samples *t*-tests showed that individuals with rural hukou had a stronger primary care employment intention compared with individuals with urban hukou (*t* = 3.42, *p* < 0.001). One-way ANOVA showed there were significant differences between groups of family income on primary care employment intention (*F* = 4.67, *p* = 0.010), with *post-hoc* tests suggesting that people from lower-income families had a stronger primary care employment intention than people from higher-income families.

**TABLE 1:** Demographic characteristics of participants (*N* = 398).

Variable	Category	n	%
Gender	Male	156	39.2
	Female	242	60.8
Year of study	Fourth year	189	47.5
	Fifth year	209	52.5
Household registration	Rural	227	57.0
	Urban	171	43.0
Parental education	College degree or above	112	28.1
	High school or below	286	71.9
Family monthly income	<3,000 RMB	89	22.4
	3,000–8,000 RMB	198	49.7
	>8,000 RMB	111	27.9
Family member in healthcare	Yes	94	23.6
	No	304	76.4

**FIGURE 2:**
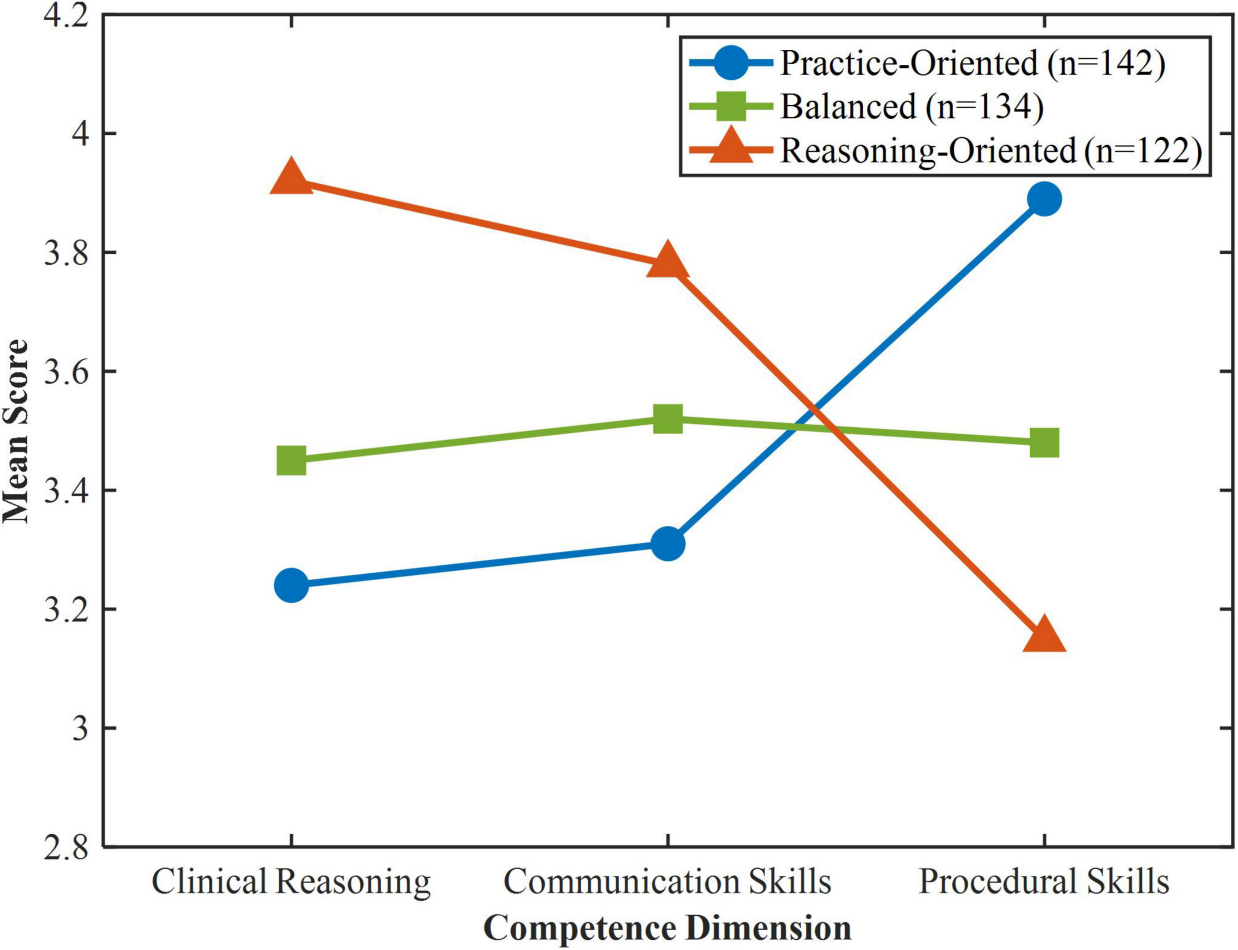
Competence characteristics across three latent profiles. Mean scores on clinical reasoning, communication skills, and procedural skills are displayed for each profile. Higher scores indicate stronger self-assessed competence in the respective dimension.

Latent profile analysis was conducted to identify distinct competence profiles based on self-ratings of participants’ skills in clinical reasoning, communication skills, and procedural skills. Models from two to five latent profiles were evaluated for comparison. The three-profile solution demonstrated the best model fit (AIC = 4831.27, BIC = 4953.42, aBIC = 4892.35, entropy = 0.84, LMR-LRT *p* = 0.023), while the four-profile model did not yield a significant improvement (LMR-LRT *p* = 0.187). As shown in Figure 2, profile 1 (*n* = 142, 35.7%) was labeled “Practice-Oriented,” characterized by relatively higher scores on procedural skills (M = 3.89) compared to clinical reasoning (*M* = 3.24) and communication skills (*M* = 3.31). Profile 2 (*n* = 134, 33.7%) was labeled “Balanced,” with moderate and relatively even scores across clinical reasoning (*M* = 3.45), communication skills (*M* = 3.52), and procedural skills (*M* = 3.48). Profile 3 (*n* = 122, 30.6%) was labeled “Reasoning-Oriented,” characterized by higher scores on clinical reasoning (*M* = 3.92) and communication skills (*M* = 3.78) but relatively lower scores on procedural skills (*M* = 3.15). Chi-square tests and one-way ANOVA revealed no significant differences across the three profiles in terms of gender, year of study, household registration, or family income.

### 3.2 Descriptive statistics and correlation analysis

The means, standard deviations, and correlation coefficients for the main variables used in this study are shown in Table 2. Medical collaboration was positively correlated with career self-efficacy, outcome expectations, and primary care employment intention, with the highest correlation with career self-efficacy. Among the three subscales, clinical teaching quality was more highly correlated with career self-efficacy than the other two, and career guidance was more highly correlated with outcome expectations. The correlation between career self-efficacy and outcome expectations was moderate, and both were positively related to primary care employment intention. Personal fulfillment was found to be more strongly related to primary care employment intention than material gain, which suggests that intrinsic rather than extrinsic motivations may have a stronger role in shaping students’ attitudes toward a career in primary care. The correlations between institutional support and other key variables were generally small in magnitude, with its association with primary care employment intention reaching significance only at the 0.05 level. Correlation patterns provided evidence supporting the hypothesized mediating pathways contained in the conceptual framework developed on the basis of SCCT. All intercorrelations among key variables were less than 0.70, and variance inflation factors were 1.18–1.67. Hence, multicollinearity was not likely to be a concern in further analysis.

**TABLE 2:** Means, standard deviations, and correlations among main study variables (*N* = 398).

Variable	M	SD	1	2	3	4	5	6	7
1. Clinical teaching quality	3.62	0.78	–						
2. Institutional support	3.48	0.81	0.47***	–					
3. Career guidance	3.57	0.76	0.52***	0.49***	–				
4. Medical educational collaboration	3.56	0.72	0.83***	0.79***	0.81***	–			
5. Career self-efficacy	3.41	0.68	0.46***	0.27**	0.38***	0.42***	–		
6. Outcome expectations	3.28	0.81	0.29**	0.22*	0.41***	0.36**	0.48***	–	
7. Primary care employment intention	2.87	0.94	0.26**	0.17*	0.32**	0.29**	0.35**	0.44***	–

*p < 0.05,

**p < 0.01,

***p < 0.001.

The comparison of main variable means across the three competence profiles is presented in Table 3. One-way ANOVA showed that there was a significant difference in career self-efficacy among the profiles, with the Reasoning-Oriented profile reporting higher scores than the Practice-Oriented profile. No significant differences were observed across profiles for medical educational collaboration or outcome expectations, suggesting that all profiles held similar perceptions of their educational experience and anticipated career outcomes. Primary care employment intention also varied significantly across profiles, with the highest mean in the Practice-Oriented group and the lowest in the Reasoning-Oriented group. These findings suggest that procedural competence orientation was associated with stronger primary care employment intention, whereas stronger clinical reasoning orientation was associated with relatively lower intention. *Post hoc* comparisons using Bonferroni correction showed that the Practice-Oriented and Reasoning-Oriented groups were significantly different in primary care employment intention, and that the Balanced group was not significantly different from the other two groups.

**TABLE 3:** Comparison of main variables across competence profiles.

Variable	Practice-oriented (*n* = 142)	Balanced (*n* = 134)	Reasoning-oriented (*n* = 122)	*F*	*p*	*Post hoc*
Medical educational collaboration	M (SD)	M (SD)	M (SD)	0.67	0.514	–
	3.52 (0.74)	3.61 (0.69)	3.54 (0.73)			
Career self-efficacy	3.29 (0.71)	3.38 (0.66)	3.58 (0.64)	6.83**	0.001	RO > PO
Outcome expectations	3.34 (0.79)	3.26 (0.82)	3.22 (0.83)	0.89	0.412	–
Primary care employment intention	3.07 (0.89)	2.84 (0.92)	2.67 (0.98)	7.14**	0.001	PO > RO

PO, Practice-Oriented; BA, Balanced; RO, Reasoning-Oriented.

**p < 0.01. *Post hoc* comparisons were conducted using Bonferroni correction.

### 3.3 Measurement model testing

Prior to testing the structural model, a confirmatory factor analysis was conducted on the measurement models of the four core constructs (Table 4). The three-factor model of medical educational collaboration, with the three dimensions of clinical teaching quality, support, and career guidance, showed a satisfactory fit, and the second-order factor model, where the three dimensions were subsumed under a second-order factor of medical educational collaboration, also showed a satisfactory fit, thereby justifying the use of the single construct of medical educational collaboration. The career self-efficacy scale was assessed using its original five-factor structure. The two items had relatively low loadings but were retained because of their importance in the scale. In regard to outcome expectations, the hypothesized three-factor structure of material returns, social recognition, and personal fulfillment had a good fit. The primary care employment intention scale, with four items forming a unidimensional latent construct, had excellent fit, with all factor loadings above 0.70. The total measurement model, with all four latent constructs, was then evaluated, and the fit indices met the criteria necessary for structural equation modeling.

**TABLE 4:** Confirmatory factor analysis results for main study variables.

Construct	χ^2^/df	CFI	TLI	RMSEA	SRMR	Factor loading range
Medical educational collaboration (three-factor)	2.34	0.94	0.92	0.058	0.047	0.64–0.85
Medical educational collaboration (second-order)	2.51	0.93	0.91	0.062	0.052	0.68–0.84
Career self-efficacy	2.67	0.92	0.90	0.065	0.054	0.58–0.81
Outcome expectations (three-factor)	2.18	0.95	0.93	0.054	0.043	0.66–0.87
Primary care employment intention	1.89	0.98	0.96	0.047	0.031	0.72–0.88
Overall measurement model	2.43	0.91	0.90	0.060	0.056	0.58–0.88

CFI, comparative fit index; TLI, Tucker-Lewis index; RMSEA, root mean square error of approximation; SRMR, standardized root mean square residual.

Convergent and discriminant validity were evaluated (Table 5). The composite reliability for all constructs exceeded the threshold of 0.70, and the average variance extracted for all constructs exceeded 0.50, ensuring the validity. The square root of AVE for all constructs exceeded the correlation value of all constructs with other constructs, ensuring discriminant validity. Overall, the results have ensured the appropriateness of the structural equation model for analysis.

**TABLE 5:** Convergent and discriminant validity of main constructs.

Construct	CR	AVE	1	2	3	4
1. Medical educational collaboration	0.88	0.56	(0.75)	(0.72)	(0.74)	(0.82)
2. Career self-efficacy	0.91	0.52	0.42			
3. Outcome expectations	0.86	0.55	0.36	0.48		
4. Primary care employment intention	0.89	0.67	0.29	0.35	0.44	

CR, composite reliability; AVE, average variance extracted. Values in parentheses along the diagonal represent the square root of AVE. Off-diagonal values represent inter-construct correlations.

### 3.4 Measurement invariance testing

Before conducting multi-group structural equation modeling, measurement invariance was assessed for the three competence profiles to ensure that the measurement of the constructs was the same for the groups (Table 6). Measurement invariance was assessed through a hierarchical series of increasingly restrictive models, including configural invariance, metric invariance, and scalar invariance.

**TABLE 6:** Measurement invariance testing across competence profiles.

Model	χ^2^	df	CFI	TLI	RMSEA	SRMR	Model comparison	Δχ^2^	Δdf	ΔCFI
Configural invariance	1247.53	531	0.912	0.901	0.058	0.054	–	–	–	–
Metric invariance	1298.67	567	0.908	0.903	0.057	0.061	Metric vs. Configural	51.14	36	0.004
Scalar invariance	1362.41	603	0.902	0.899	0.059	0.063	Scalar vs. Metric	63.74*	36	0.006
Partial scalar invariance	1319.28	597	0.906	0.902	0.057	0.062	Partial scalar vs. Metric	20.61	30	0.002

*p < 0.05. ΔCFI values below 0.010 indicate invariance is supported.

The configural invariance model was found to fit the data adequately and allowed the factor loadings and intercepts to be freely estimated across groups while equating the factor structure. This result shows that the same factor structure is maintained across the three competence profiles. The metric invariance model equated the factor loadings across groups. The change in CFI between the configural and metric model was 0.004, which is less than the cut-off point of 0.010. Moreover, the chi-square difference test was also found to be insignificant. The results provided evidence of metric invariance.

The scalar invariance constraint required equality of item intercepts across groups. The chi-square difference test between the scalar and metric models was significant, with a decrease in CFI of 0.006. Although ΔCFI was less than 0.010, modification indices were examined to identify specific locations of possible non-invariant values. Two item intercepts within the career self-efficacy scale exhibited notably larger discrepancies across the three competence profiles than the remaining items, suggesting that students with distinct competence orientations may differentially interpret certain aspects of career decision-making. A partial scalar invariance model was estimated by setting these two intercepts to be free to differ across groups and constraining the remaining 23 intercepts to be equal. This model provided a better fit than the full scalar model, as the chi-square difference between the partial scalar and metric models was non-significant (ΔCFI = 0.002). Partial scalar invariance was therefore established and considered sufficient to conduct multi-group structural model and latent mean difference tests.

### 3.5 Structural model analysis

**FIGURE 3:**
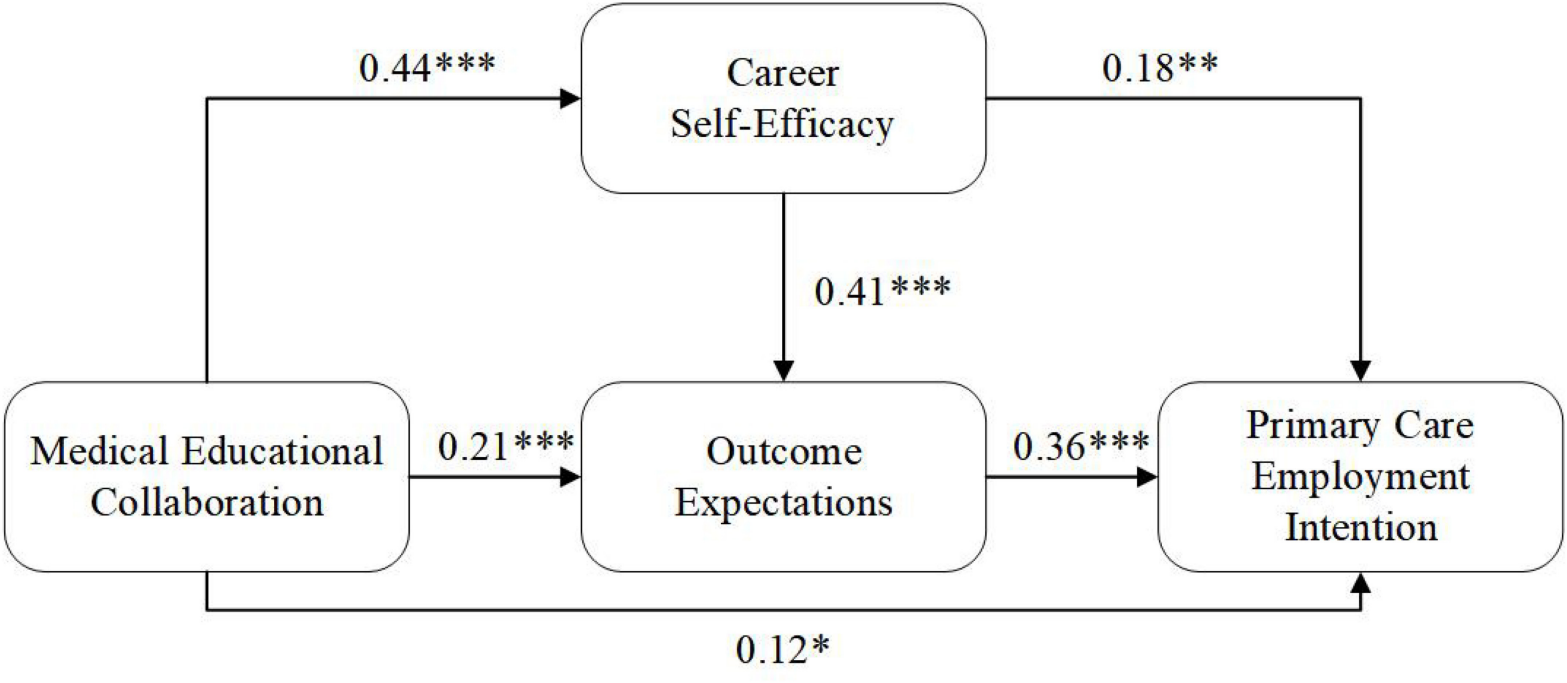
Path coefficients of the structural model. *p < 0.05, **p < 0.01, ***p < 0.001.

With measurement invariance established, the structural model was then evaluated to determine if it supported relationships among medical educational collaboration, career self-efficacy, outcome expectations, and primary care employment intention. The structural model had acceptable fit (χ^2^/df = 2.38, CFI = 0.91, TLI = 0.90, RMSEA = 0.059, SRMR = 0.058). The standardized path coefficients are presented in Figure 3.

Medical educational collaboration was significantly and positively associated with career self-efficacy (β = 0.44, *p* < 0.001), representing a medium-to-large association, and with outcome expectations (β = 0.21, *p* < 0.001), representing a small-to-medium association. Career self-efficacy was positively associated with outcome expectations (β = 0.41, *p* < 0.001) and primary care employment intention (β = 0.18, *p* = 0.003), the latter representing a small association. Outcome expectations showed a significant positive association with primary care employment intention (β = 0.36, *p* < 0.001), representing a medium association. The direct association between medical educational collaboration and primary care employment intention was small but statistically significant (β = 0.12, *p* = 0.027), indicating partial mediation.

The mediation effects were examined using bias-corrected bootstrap confidence intervals with 5,000 resamples. The total indirect association of medical educational collaboration with primary care employment intention was significant (β = 0.23, 95% CI [0.168, 0.297]). Three specific indirect pathways were identified: through career self-efficacy alone (β = 0.08, 95% CI [0.032, 0.136]), through outcome expectations alone (β = 0.08, 95% CI [0.034, 0.131]), and sequentially through both mediators (β = 0.07, 95% CI [0.041, 0.103]). The total association between medical educational collaboration and primary care employment intention was 0.35 (95% CI [0.248, 0.452]), with approximately 66% of this association transmitted through indirect pathways.

### 3.6 Multi-group comparison analysis

Multi-group structural equation modeling was conducted to evaluate the equality of the structural paths on the three competence profiles. The unconstrained model, which allowed all the structural paths to differ freely among the groups, demonstrated acceptable fit (χ^2^/df = 2.21, CFI = 0.90, TLI = 0.89, RMSEA = 0.062, SRMR = 0.064). The constrained model, which fixed all structural paths to be equal across groups, showed a significant decrease in model fit compared to the unconstrained model (Δχ^2^ = 38.72, Δdf = 12, *p* < 0.01), indicating that at least some structural paths differed significantly across competence profiles. In order to identify which of the paths were different across groups, a series of partial invariance tests were performed by progressively fixing individual paths equal to each other. There were three paths that were significantly different across groups: medical educational collaboration to career self-efficacy (Δχ^2^ = 11.34, Δdf = 2, *p* < 0.01), career self-efficacy to primary care employment intention (Δχ^2^ = 9.87, Δdf = 2, *p* < 0.01), and outcome expectations to primary care employment intention (Δχ^2^ = 8.56, Δdf = 2, *p* < 0.05). The remaining paths did not differ significantly across groups.

**FIGURE 4:**
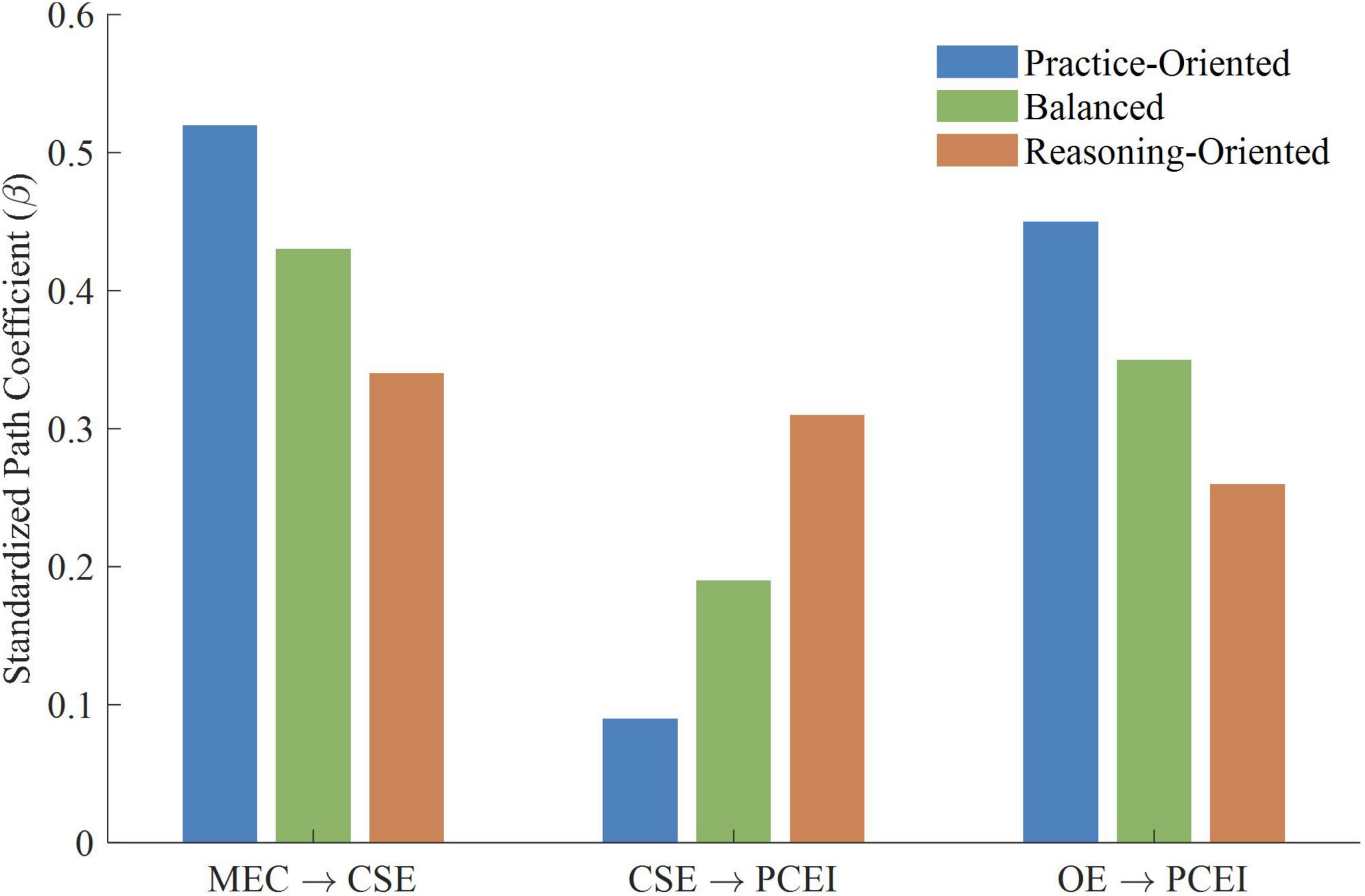
Comparison of path coefficients across competence profiles. MEC, Medical Educational Collaboration; CSE, Career Self-Efficacy; OE, Outcome Expectations; PCEI, Primary Care Employment Intention.

As shown in Figure 4, the three competence profiles showed a unique course on these paths of differential functioning. For the path from medical educational collaboration to career self-efficacy, the Practice-Oriented profile showed the strongest association (β = 0.52, *p* < 0.001), followed by the Balanced profile (β = 0.43, *p* < 0.001) and the Reasoning-Oriented profile (β = 0.34, *p* < 0.001), representing large, medium-to-large, and medium associations, respectively. However, the association between career self-efficacy and primary care employment intention was patterned differently: the Reasoning-Oriented profile demonstrated the strongest association (β = 0.31, *p* < 0.001), representing a medium association, followed by the Balanced profile (β = 0.19, *p* = 0.018), representing a small association, while the Practice-Oriented profile showed a non-significant association (β = 0.09, *p* = 0.241). For the association between outcome expectations and primary care employment intention, the Practice-Oriented profile exhibited the strongest association (β = 0.45, *p* < 0.001), representing a medium-to-large association, followed by the Balanced profile (β = 0.35, *p* < 0.001), representing a medium association, whereas the Reasoning-Oriented profile showed the weakest association (β = 0.26, *p* < 0.001), representing a small-to-medium association. These findings suggest that students with different competence profiles may rely on different psychological pathways when forming primary care employment intention.

The indirect associations of medical educational collaboration with primary care employment intention also differed among competence profiles. For the Practice-Oriented profile, the indirect association through outcome expectations (β = 0.10, 95% CI [0.048, 0.171]) was the only statistically significant indirect pathway, while the indirect association through career self-efficacy failed to reach statistical significance (β = 0.05, 95% CI [−0.019, 0.116]). In contrast, for the Reasoning-Oriented profile, the indirect association through career self-efficacy (β = 0.11, 95% CI [0.052, 0.178]) was the relatively stronger indirect pathway, exceeding the indirect association through outcome expectations (β = 0.05, 95% CI [0.014, 0.098]). The Balanced profile showed relatively equal contributions from both pathways (β = 0.08 and β = 0.07, respectively). These findings suggest that the associations between medical educational collaboration and primary care employment intention may operate through different indirect pathways depending on students’ competence profiles.

## 4 Discussion

The overall structural model confirmed that associations between collaboration in medical education and primary care employment intention operated through both direct and indirect pathways, where career self-efficacy and outcome expectations acted as parallel and serial mediators. This result supports the theoretical assumptions proposed by Lent et al. (12), which argue that learning experiences shape career intentions through cognitive-person variables. The mediating role of career self-efficacy is supported by Chen et al. (16), who demonstrated in their study that career-related interventions were associated with students’ career decision confidence. The important role of outcome expectations and the preponderant role played by personal fulfillment in comparison to material rewards is supported by Pfarrwaller et al. (27), who noted in their study among medical students that altruistic motivations were more significantly associated with career aspirations in primary care than material rewards.

Three competence profiles were discovered through latent profile analysis, thus adding to existing research that has mainly employed variable-centered strategies. Person-centered strategies may detect population heterogeneity that could be obscured through common regression analyses (28), and the three-profile solution discovered in this study is consistent with trends discovered in previous research on competence heterogeneity among medical trainees. Additionally, the multi-group analysis suggested that three pathways from SCCT operated differently across the three competence profiles.

The students in the Practice-Oriented profile were the ones whose career self-efficacy showed the strongest association with collaboration in medical education. However, the association between career self-efficacy and primary care employment intention was found to be non-significant for this group. Rather, outcome expectations were found to be the key factor associated with primary care employment intention. Contrary to the conventional assumption that self-efficacy universally drives career choice (14), Practice-Oriented students exhibited the highest primary care employment intention despite reporting relatively lower career self-efficacy. For these students, the opportunity to apply procedural skills in primary care settings may be associated with their stronger employment intention through outcome-related considerations rather than through efficacy beliefs, which is in line with prior evidence that medical career selection involves an assessment of the fit between perceived skills and specialty characteristics (29).

The students in the Reasoning-Oriented profile showed a contrasting pattern where career self-efficacy was the relatively stronger mediating pathway. Although the association between medical educational collaboration and career self-efficacy was relatively weaker for these students, the association between career self-efficacy and primary care employment intention was the strongest among the three profiles. These students, who reported higher skills in clinical reasoning and communication, appear to rely more on career confidence in forming primary care employment intention. Their relatively lower primary care employment intention may be associated with perceptions of primary care as insufficiently challenging for their analytical capabilities, which parallels findings by Leutritz et al. (10) that specialty choices among medical students are associated with perceived intellectual stimulation. The results for the Balanced profile showed relatively equal contributions from both pathways, which may tentatively suggest that students with moderate and relatively even levels of competency could benefit from comprehensive programs that simultaneously target efficacy beliefs and outcome expectations.

These differentiated pathways tentatively suggest that a uniform strategy for delivering career guidance may not be the most effective. For Practice-Oriented students, career guidance programs that emphasize the practical value of primary care work, such as structured community immersion experiences and exposure to the tangible contributions that primary care physicians make to community health, may strengthen outcome expectations toward primary care. For Reasoning-Oriented students, who appear to rely more heavily on career self-efficacy in forming primary care employment intention, interventions aimed at enhancing career confidence may be worth exploring. These could include case-based learning activities that highlight the diagnostic complexity and intellectual demands of primary care, which may help reframe students’ perceptions of primary care as a cognitively stimulating field (10). For Balanced students, comprehensive career guidance programs that simultaneously address both self-efficacy beliefs and outcome expectations, such as mentorship programs pairing students with primary care physicians and structured career planning workshops, may be appropriate given the relatively equal contribution of both pathways observed in this group. The heterogeneity of the patterns found in the profiles points to the potential value of career counseling programs based on competencies that adapt intervention strategies to students’ profiles, which is in line with the latest findings that indicate that students with different profiles need distinct strategies (30). The effectiveness of these differentiated career guidance strategies warrants empirical validation through future intervention studies.

Several limitations should be acknowledged. Because this is a cross-sectional study relying on a single-source self-report design, it is impossible to make inferences regarding the causal nature of the observed associations, and the use of self-report measures may introduce the risk of social desirability bias, particularly regarding primary care employment intention, as students may overestimate their willingness to work at the grassroots level. Although Harman’s single-factor test was conducted and did not indicate a substantial concern, this procedure has recognized limitations as a diagnostic tool and may not fully capture all sources of common method variance; future studies are encouraged to adopt more rigorous procedural and statistical remedies, such as temporally separated measurements or the inclusion of marker variables. Potential endogeneity concerns cannot be ruled out, as unobserved variables such as prior clinical exposure or academic performance may confound the observed associations. These limitations could be addressed in future longitudinal studies. The population sampled was exclusively from Yunnan Province, and thus the findings should be interpreted within this specific geographic and institutional context, and generalization to other provinces with different socioeconomic environments and healthcare systems should be approached with caution. The classification of competence profiles was conducted on the basis of self-perceived rather than objectively assessed clinical competency, and several scales used in the present study, including the medical educational collaboration scale, the outcome expectations scale, the primary care employment intention scale, and the clinical competence self-assessment, were either developed specifically for this study or adapted from existing theoretical frameworks based on the Chinese educational context. Although these instruments demonstrated satisfactory internal reliability and construct validity in the present sample, their psychometric properties have yet to be established through independent external validation, and cross-sample replication is warranted to confirm their generalizability. The outcome variable is limited to self-reported primary care employment intention rather than actual career behavior, and future research incorporating longitudinal behavioral follow-up would strengthen the validity of these findings.

## 5 Conclusion

This study examined the differential associations between collaboration in medical education and primary care employment intention among medical students in Yunnan Province, China, with different competence profiles. Based on Social Cognitive Career Theory, this study used latent profile analysis to derive three competence profiles and multi-group structural equation modeling to test pathway heterogeneity. The results show that the associations between collaboration in medical education and primary care employment intention operated through career self-efficacy and outcome expectations, with about 66% of the total association operating through the indirect paths. The multi-group analysis showed that these SCCT-based pathways varied across the competence profiles: for the Practice-Oriented students, the outcome expectations pathway was found to be the more prominent mediator, whereas for the Reasoning-Oriented students, the career self-efficacy pathway played a relatively more central role, and for the Balanced profile, the two pathways contributed relatively equally. These findings provide empirical evidence suggesting that SCCT-based pathways may vary as a function of individual competence characteristics, contributing to the person-centered perspective in career development research, and highlight the need for differentiated approaches to providing career guidance strategies by medical education organizations to enhance the primary care workforce in western China.

## Data Availability

The original contributions presented in this study are included in the article/supplementary material, further inquiries can be directed to the corresponding author.
